# Association between serum 25-hydroxyvitamin D concentrations and platelet to high-density lipoprotein cholesterol ratio: evidence from two population-based studies

**DOI:** 10.3389/fnut.2025.1662753

**Published:** 2025-10-06

**Authors:** Ming Wang, Zhi-Long Huang, Cong-Liang Rao, Xing-Shu Zhu, Bei-Jing Cheng, Jun Zhu

**Affiliations:** ^1^The Lu’an Hospital Affiliated to Anhui Medical University, Lu’an, Anhui, China; ^2^The Lu’an People's Hospital, Lu’an, Anhui, China; ^3^Key Laboratory of Environmental Medicine Engineering, Ministry of Education, School of Public Health, Southeast University, Nanjing, Jiangsu, China

**Keywords:** vitamin D, 25-hydroxyvitamin D, PHR, platelet to high-density lipoprotein cholesterol ratio, combined datasets

## Abstract

**Background:**

The platelet to high-density lipoprotein cholesterol ratio (PHR) is an emerging marker of inflammation and metabolic health, combining platelet counts and HDL cholesterol (HDL-C) levels. Vitamin D is essential for various physiologic processes, including immune modulation and lipid metabolism. Our study investigates the association between serum 25-hydroxyvitamin D [25(OH)D] concentrations and PHR.

**Methods:**

We conducted cross-sectional analyses of two population-based datasets: NHANES (*n* = 36,238) from the U.S. and the baseline survey of a Chinese cohort study (*n* = 1,122). Serum 25(OH)D and blood PHR were assessed, with PHR defined as the ratio of platelet count to HDL-C (mmol/L). To examine the associations of 25(OH)D with PHR, we used weighted linear regression models and weighted restricted cubic splines (RCS), adjusting for potential confounders. Additionally, stratified analyses were performed based on potential influencing factors.

**Results:**

After stepwise adjusting for cycles, demographic characteristics, lifestyle factors, and health conditions (including medication use), survey-weighted linear regression analysis of the NHANES database identified a significant negative association of serum 25(OH)D levels with PHR. Specifically, for every 1-unit increase in 25(OH)D, PHR decreased by 0.23 to 0.41 units across models. This association remained significant when comparing the highest quartile (Q4) to the lowest quartile (Q1) of 25(OH)D, with PHR decreasing by 14.84 to 27.65 units across models. RCS analysis further supported a linear negative association of 25(OH)D with PHR. Similar results were observed for 25-hydroxyvitamin D_3_ [25(OH)D_3_]. Furthermore, analyses in the Chinese population confirmed the inverse association between serum total 25(OH)D and PHR. Notably, the stronger association observed in females was consistent across both populations, with statistically significant interaction effects.

**Conclusion:**

Our study found that serum 25(OH)D levels were significantly negatively correlated with PHR, particularly in females. These results suggest that 25(OH)D may help modulate PHR, with potential implications for disease prevention. Future research should confirm causality and explore underlying mechanisms.

## 1 Introduction

The platelet to high-density lipoprotein cholesterol ratio (PHR), which combines platelet counts and HDL cholesterol (HDL-C) levels, has emerged as a novel biomarker of inflammation and metabolic health (1, 2). PHR was originally proposed by Jialal et al. (1) as a new biomarker to predict metabolic syndrome. Recently, the predictive value of the PHR has been recognized not only for metabolic syndrome, but also for its potential in diagnosing and predicting a range of other disorders, including cardiovascular disease, kidney stones, non-alcoholic fatty liver disease, and depression (3–6). Taken together, these findings suggest that PHR is a promising biomarker with potential clinical relevance across various health issues.

The concept of PHR has emerged in recent years, with most studies focusing on its relationship with disease (3–6), while relatively few have examined the factors that influence PHR. Investigating these influencing factors, particularly modifiable factors, is crucial, as they can modulate PHR levels and potentially help prevent or mitigate the onset and progression of certain diseases. We reviewed risk factors associated with platelet counts and HDL-C levels, including environmental and lifestyle (7–9). Nutrition has been recognized as a simple and effective modulator of various biomarkers in multiple studies (10–12), and perhaps a key factor influencing PHR levels.

Vitamin D is an essential micronutrient involved in numerous physiological processes, including calcium-phosphorus metabolism, immune modulation, and lipid metabolism (13–17). A review examined the effects of vitamin D on inflammatory signaling, coagulation mechanisms, and endothelial cell function, highlighting the important role of vitamin D in vascular health and immune regulation (18). A few epidemiological evidence also suggested a potential link between vitamin D insufficiency and increased platelet counts (19, 20). Several reviews have explored the impact of vitamin D on lipid metabolism, emphasizing its effects on cholesterol and triglyceride regulation, as well as its importance in cardiovascular health (21, 22). For instance, both Faridi et al. (23) and Lupton et al. (17) identified a strong relationship between vitamin D deficiency and reduced HDL-C levels. In conclusion, vitamin D is essential for the regulation of inflammation and lipid metabolism, and deficiency leads to elevated platelet counts and dyslipidemia.

To date, most research has focused on the associations between vitamin D and platelet count or HDL levels. Recently, a study using multivariate linear regression reported a negative association between vitamin D and PHR based on NHANES data (24). However, that study offered limited exploration of differences among specific population subgroups and lacked external validation in independent population, which may affect the robustness and generalizability of the findings. Therefore, to address these gaps, the present study builds upon previous work by conducting a more comprehensive investigation of the relationship between serum 25-hydroxyvitamin D [25(OH)D] concentrations and PHR, including extensive subgroup analyses and external validation across two independent populations. As a metabolite of vitamin D, 25(OH)D serves as the primary indicator of vitamin D status in the bloodstream (25).

## 2 Materials and methods

### 2.1 Study population

**Figure 1:**
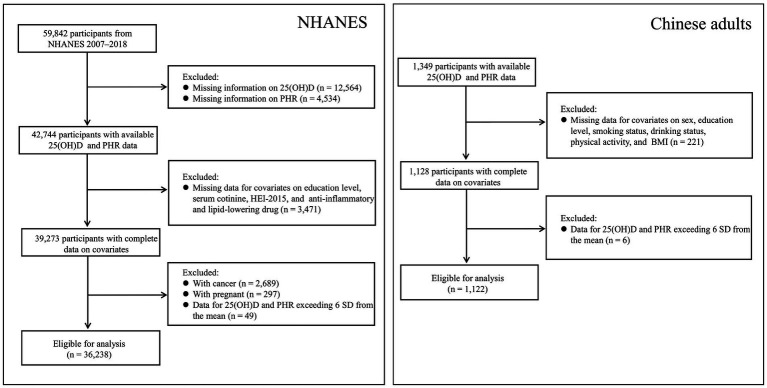
Participant selection flowchart from NHANES and Chinese adults.

Given the availability of data and the influence of the COVID-19 pandemic, the analysis was limited to the NHANES 2007–2018 cycles, as 25-hydroxyvitamin D_3_ [25(OH)D_3_] data became available starting in 2007. NHANES, a nationwide survey conducted every 2 years, assesses the health and nutrition of the U.S. population through questionnaires, physical exams, and biospecimen analysis. The U.S. Centers for Disease Control and Prevention provides a detailed overview of the study methodology.^1^ In this study, we included participants with complete data on 25(OH)D concentrations, PHR, and selected covariates but excluded pregnant women and patients with cancer. We also excluded data for 25(OH)D and PHR values that deviated more than 6 standard deviations (SD) from the mean (*n* = 49). A flowchart summarizing the inclusion and exclusion criteria for study participants is presented in Figure 1. Ethical approval for the NHANES was granted by the National Center for Health Statistics. All participants provided written informed consent before taking part in the survey.

The Chinese data used in this study were derived from the baseline survey of a cohort study, which was initiated in Fuyang City, Anhui Province, China, between July and September 2018. All participants were required to complete a corresponding self-designed questionnaire. For the present analysis, those with complete serum 25(OH)D and PHR data (*n* = 1,349) were selected. After excluding individuals with missing data on selected covariates (*n* = 221) and those exceeding ±6 SD from the mean for either 25(OH)D or PHR (*n* = 6), a total of 1,122 participants were included in the final analysis (Figure 1). Ethical approval for this study was obtained from the Ethics Committee of the Anhui Medical University (No. 20190288), and written informed consent was obtained from all participants prior to their enrollment.

### 2.2 Assessment of serum 25(OH)D

In the NHANES dataset, ultra-high performance liquid chromatography–tandem mass spectrometry (UHPLC–MS/MS) was utilized for the precise quantification of 25(OH)D_3_ and 25-hydroxyvitamin D_2_ [25(OH)D_2_] in human serum. Concentrations of 25(OH)D below the limit of detection (LOD) were assigned a value of LOD divided by the square root of two. Total 25(OH)D is calculated by adding 25(OH)D_2_ and 25(OH)D_3_ concentrations. In this study, the detection rate for 25(OH)D_2_ was 17.62%, with relatively low concentrations, indicating that 25(OH)D_3_ predominantly determines the total 25(OH)D concentrations. Therefore, subsequent analyses focused primarily on Total 25(OH)D and 25(OH)D_3_.

In our Chinese population, serum total 25(OH)D levels were measured using commercial chemiluminescence immunoassay kits (DiaSorin, Stillwater, MN, United States) by well-trained researchers. The coefficients of variation (CV) for both intra- and inter-assay precision were less than 10%.

### 2.3 Assessment of PHR

Blood count and lipid profile data were obtained from the laboratory records of NHANES and from the laboratory database of the Chinese population. PHR was calculated using the platelet (PLT) count and HDL-C. PLT count was expressed as 1,000 cells/μL, HDL-C was measured in mmol/L in both databases, and PHR was calculated using the same formula in both cohorts (26):


PHR=PLTcount(1000cells/uL)/HDL−C(mmol/L)

### 2.4 Covariates

Potential confounders were identified based on previous studies of lipid profiles and PLT (7, 8, 27). The covariates from NHANES were classified into four categories: cycles, demographic characteristics (age, sex, race, education level, poverty-to-income ratio (PIR), and marital status), lifestyle factors (serum cotinine, drinking status, physical activity, and the Healthy Eating Index 2015 (HEI-2015)), and health conditions and medication use (body mass index (BMI), diabetes, hypertension, and the use of anti-inflammatory or lipid-lowering medications). The PIR was calculated by dividing the midpoint of the reported family income category by the poverty threshold established by the U.S. Census Bureau for the corresponding calendar year (25). Based on this ratio, PIR was classified into three groups: low (0–1.3; reference), middle (1.31–3.50), and high (>3.50). Smoking status was determined based on serum cotinine levels, with concentrations exceeding 14 ng/mL classified as smokers, while those at or below this threshold were considered non-smokers (28). Physical activity levels were assessed using average metabolic equivalent task (MET) scores, with intensity categories defined as moderate for activities at ≥4 METs and vigorous for those at ≥8 METs. The total physical activity score was derived by aggregating the weekly MET-minutes across both moderate and vigorous activity levels. Physical activity levels were classified as “low” for <600 MET-minutes per week, “moderate” for 600–1,200 MET-minutes per week, and “optimal” for ≥1,200 MET-minutes per week (29). Dietary quality was assessed based on the 2015–2020 Dietary Guidelines for Americans. Scores were classified as “optimal” (≥70), “average” (50–70), and “inadequate” (<50). BMI was calculated by dividing body weight (kg) by the square of height (m^2^). It was categorized into three groups: “normal or underweight” (<25.0 kg/m^2^; reference), “overweight” (25.0–29.9 kg/m^2^), and “obese” (≥30.0 kg/m^2^). Hypertension was defined as a self-reported diagnosis, the use of antihypertensive medications, a systolic blood pressure >140 mmHg, or a diastolic blood pressure >90 mmHg. Diabetes was defined by a self-reported diagnosis, the use of insulin or oral hypoglycemic agents, a fasting plasma glucose level ≥126 mg/dL, or a hemoglobin A1c level ≥6.5%. The use of anti-inflammatory or lipid-lowering medications was based on self-report.

Covariates in the Chinese dataset were selected to align with those used in NHANES and were obtained through a structured, self-designed questionnaire and physical measurements. Economic status was self-reported and classified into three categories: poverty, average or wealthy, and missing; the latter included individuals with unclear or missing responses due to a high non-response rate. BMI was categorized according to Chinese guidelines: normal or underweight (<24.0 kg/m^2^), overweight (24.0–27.9 kg/m^2^), and obese (≥28.0 kg/m^2^). Diabetes and hypertension were defined based on self-reported physician diagnoses, current medication use, or relevant clinical indicators, in accordance with the definitions used in NHANES.

### 2.5 Statistical analysis

#### 2.5.1 Analysis of NHANES data

Since no unique weights were available for 25(OH)D concentrations and PHR, mobile examination center sample weights were applied in the estimation calculations, in accordance with NHANES guidelines. In the present study, variables with a normal or near-normal distribution were presented as weighted mean ± standard error (mean ± SE). Between-group comparisons were performed using weighted *t*-tests and weighted analysis of variance (ANOVA). Categorical variables were presented as unweighted frequencies and weighted proportions (*n*, %) (30). Between-group comparisons were conducted using weighted χ^2^ test.

Survey-weighted linear regression models were utilized to assess the relationship between 25(OH)D and PHR. In the regression models, 25(OH)D was analyzed as both a continuous and categorical variable (quartiles), with the lowest quartile designated as the reference group. Additionally, we conducted trend analysis (p-trend) to evaluate dose-response relationships. We constructed four models, progressively adjusting for the following covariates: cycles, demographic characteristics, lifestyle factors, and health conditions (including medication use). We also used weighted restricted cubic splines (RCS) to explore the relationship between 25(OH)D and PHR. Two strategies were employed for handling missing covariate values: if the missing data rate was less than 5%, the corresponding records were omitted (Figure 1); otherwise, the missing values were classified as “missing” in the analysis.

To assess the robustness of the relationship between 25(OH)D and PHR and to identify potentially vulnerable subgroups, a subgroup analysis was conducted based on demographic characteristics, lifestyle factors, and health conditions (including medication use). We performed three sensitivity analyses to assess the reliability of our findings. First, we applied general linear regression to evaluate the relationship between 25(OH)D and PHR. Second, we categorized PHR into a binary variable using the median as the cutoff point and performed both weighted logistic regression and weighted RCS to further explore the relationship. Finally, we categorized 25(OH)D_2_ as a binary variable using the LOD as the cutoff point and applied survey-weighted linear regression to assess the association. All statistical analyses were conducted using R (version 4.1.3). A two-tailed *p* < 0.05 was considered statistically significant.

#### 2.5.2 Analysis of data from the Chinese population

The statistical analysis followed the same procedures as those applied to the NHANES data, except that sampling weights were not used. All statistical analyses were conducted using R (version 4.1.3). A two-tailed *p* < 0.05 was considered statistically significant.

## 3 Results

### 3.1 Baseline characteristics

This study included 36,238 participants, representing the approximately 217.9 million noninstitutionalized population residing in the United States (Table 1). This study included participants across a wide age range, with the distribution as follows: 7.52% were under 12 years of age, 12.64% were between 12 and 19 years, 61.88% were between 20 and 59 years, and 17.96% were 60 years or older. The sex distribution was balanced, with 50.07% female and 49.93% male. The majority of the population (63.4%) identified as Non-Hispanic White. More detailed information on baseline characteristics is provided in Table 1. Additionally, the histogram revealed approximately normal distributions of PHR and serum 25(OH)D levels (Supplementary Figure S1). The demographics of this study population were similar to those of the original NHANES population, which included completed data on serum 25(OH)D levels and PHR (Supplementary Table S1).

**Table 1:** The characteristics of all eligible participants from NHANES.

Variables	Total	PHR		*p* value
	*n* (%)	Mean	SE	
Cycles				<0.001
2007–2008	5,559 (14.46)	219.18	2.28	
2009–2010	6,858 (16.89)	196.65	2.05	
2011–2012	5,944 (17.01)	190.87	2.10	
2013–2014	6,318 (17.15)	191.09	2.23	
2015–2016	6,069 (17.17)	189.04	2.57	
2017–2018	5,490 (17.32)	194.06	3.07	
Age (year)				<0.001
<12	4,958 (7.52)	215.96	1.72	
12–19	6,158 (12.64)	204.04	1.48	
20–59	17,632 (61.88)	198.59	1.16	
≥60	7,490 (17.96)	174.26	1.52	
Sex				<0.001
Female	18,133 (50.07)	190.70	1.19	
Male	18,105 (49.93)	201.75	1.06	
Race/ethnicity				<0.001
Mexican American	6,685 (10.46)	212.05	1.84	
Other Hispanic	4,027 (6.43)	208.19	1.70	
Non-Hispanic Black	7,961 (11.60)	191.56	1.29	
Non-Hispanic White	13,128 (63.44)	193.02	1.36	
Other race/multiracial	4,437 (8.06)	198.02	2.17	
Education level				<0.001
Below high school	16,275 (30.70)	187.11	1.19	
High school	6,317 (19.88)	201.28	1.61	
Above high school	13,646 (49.42)	207.61	1.07	
PIR				<0.001
Low (0–1.3)	12,105 (22.78)	199.17	1.11	
Middle (1.31–3.50)	12,263 (33.54)	186.99	1.50	
High (>3.50)	8,859 (37.02)	207.64	1.47	
Missing	3,011 (6.66)	193.58	2.04	
Marital status				<0.001
Never married	4,878 (15.54)	208.45	1.31	
Married or cohabit	14,962 (50.61)	193.14	1.23	
Widowed/divorced/separated	5,276 (13.67)	196.92	1.78	
Missing	11,122 (20.18)	188.76	1.56	
Serum cotinine				<0.001
<14 ng/ml	29,530 (79.48)	193.67	1.04	
≥14 ng/mL	6,708 (20.52)	206.09	1.52	
Drinking status				<0.001
Never	3,621 (8.58)	208.90	2.13	
Former	3,463 (8.97)	205.56	1.22	
Now	16,825 (58.27)	197.03	2.17	
Missing	12,329 (24.18)	190.27	1.16	
Physical activity				<0.001
Low	3,982 (11.40)	191.50	1.17	
Moderate	16,362 (52.13)	195.18	1.81	
Optimal	3,227 (9.50)	193.30	1.79	
Missing	12,667 (26.97)	206.79	1.25	
HEI-2015				<0.001
Inadequate	19,554 (53.45)	189.35	1.21	
Average	13,977 (38.60)	205.15	1.08	
Optimal	2,707 (7.95)	169.56	1.94	
BMI				<0.001
Normal or low weight	15,612 (38.94)	176.61	0.94	
Overweight	9,805 (28.99)	193.84	1.21	
Obese	10,821 (32.07)	222.18	1.35	
Diabetes				<0.001
No	32,745 (91.92)	194.86	1.01	
Yes	3,493 (8.08)	211.62	2.34	
Hypertension				<0.001
No	24,491 (69.70)	222.27	2.33	
Yes	10,039 (27.72)	194.38	1.11	
Missing	1708 (2.58)	198.41	1.37	
Anti-inflammatory drug				0.002
No	31,426 (86.42)	196.88	1.03	
Yes	4,812 (13.58)	192.03	1.72	
Lipid-lowering drug				0.410
No	34,757 (95.83)	196.12	1.00	
Yes	1,481 (4.17)	198.40	2.97	
Total 25(OH)D (nmol/L)				<0.001
Q1 (6.15–45.0)	9,098 (18.47)	209.35	1.42	
Q2 (45.1–60.3)	9,054 (21.69)	207.06	1.33	
Q3 (60.4–76.4)	9,030 (26.41)	198.13	1.41	
Q4 (76.5–215)	9,056 (33.42)	180.42	1.33	
25(OH)D_3_ (nmol/L)				<0.001
Q1 (3.72–41.7)	9,073 (18.59)	207.59	1.33	
Q2 (41.8–57.4)	9,106 (21.72)	207.32	1.45	
Q3 (57.5–73.4)	9,001 (25.97)	198.32	1.36	
Q4 (73.5–214)	9,058 (33.72)	181.18	1.34	

PHR, platelet to high-density lipoprotein cholesterol ratio; SE, standard error; PIR, poverty-to-income ratio; HEI, healthy eating index; BMI, body mass index. Continuous variables were expressed as weighted means ± SEs and categorical variables were described as *n* (%). n: numbers of subjects; %: weighted percentage. Total 25(OH)D: the combined concentrations of 25(OH)D₂ and 25(OH)D₃.

The differences in PHR across the various groups are also presented in Table 1. Higher PHR levels were observed in participants who were younger, male, of Mexican American or other Hispanic descent, more highly educated, had higher income, and were never married. These individuals were also more likely to be smokers, non-drinkers, engage in moderate physical activity, have average HEI-2015 scores, be obese, have diabetes, be free of hypertension, and not use anti-inflammatory medications.

### 3.2 Associations between serum 25(OH)D levels and PHR

Table 2 presents the associations of serum 25(OH)D levels with PHR using survey-weighted linear regression. After adjusting for cycles, we observed a negative association between total 25(OH)D (as a continuous variable) and PHR. We then performed stepwise adjustments for demographic characteristics, lifestyle factors, and health conditions (including medication use). After these adjustments, the negative association remained statistically significant. For each 1-unit increase in 25(OH)D, PHR decreased by 0.23 to 0.41 units.

**Table 2:** Associations of serum 25(OH)D concentrations with PHR using weighted linear regression from NHANES.

Characteristics	Continuous	Q1	Q2	Q3	Q4	*P* for trend
	*β* (95% *CI*)	Reference	*β* (95% *CI*)	*β* (95% *CI*)	*β* (95% *CI*)	
Total 25(OH)D						
Model 1	−0.47 (−0.51, −0.43)	1.00	−1.75 (−4.71, 1.21)	−11.23 (−14.43, −8.03)	−28.64 (−31.64, −25.64)	<0.001
Model 2	−0.41 (−0.46, −0.37)	1.00	−6.00 (−9.11, −2.89)	−14.92 (−18.17, −11.66)	−27.65 (−31.00, −24.31)	<0.001
Model 3	−0.35 (−0.40, −0.31)	1.00	−4.41 (−7.44, −1.37)	−12.54 (−15.81, −9.26)	−23.56 (−26.99, −20.12)	<0.001
Model 4	−0.23 (−0.27, −0.18)	1.00	−2.16 (−5.24, 0.92)	−7.13 (−10.09, −4.17)	−14.84 (−18.03, −11.66)	<0.001
25(OH)D_3_						
Model 1	−0.45 (−0.49, −0.41)	1.00	0.16 (−3.10, 3.42)	−9.15 (−12.19, −6.10)	−26.08 (−29.14, −23.03)	<0.001
Model 2	−0.41 (−0.45, −0.37)	1.00	−5.05 (−8.23, −1.87)	−14.27 (−17.25, −11.29)	−26.62 (−29.72, −23.52)	<0.001
Model 3	−0.35 (−0.39, −0.31)	1.00	−3.44 (−6.64, −0.24)	−11.79 (−14.88, −8.70)	−22.46 (−25.68, −19.24)	<0.001
Model 4	−0.21 (−0.25, −0.17)	1.00	−1.18 (−4.21, 1.84)	−6.37 (−9.19, −3.55)	−13.29 (−16.20, −10.38)	<0.001

PHR, platelet to high-density lipoprotein cholesterol ratio; CI, confidence interval; Q1, the first quartile; Q2, the second quartile; Q3, the third quartile; Q4, the fourth quartile. Total 25(OH)D: the combined concentrations of 25(OH)D₂ and 25(OH)D₃. Model 1: was adjusted for cycles. Model 2: cycles and demographic characteristics (age, sex, race, education level, PIR, and marital status). Model 3: further adjusted for lifestyle factors (serum cotinine, drinking status, physical activity, and HEI-2015) in addition to the adjustments in Model 2. Model 4: further adjusted for health conditions and medication use (BMI, diabetes, hypertension, anti-inflammatory drugs, and lipid-lowering drugs) in addition to the adjustments in Model 3.

**Figure 2:**
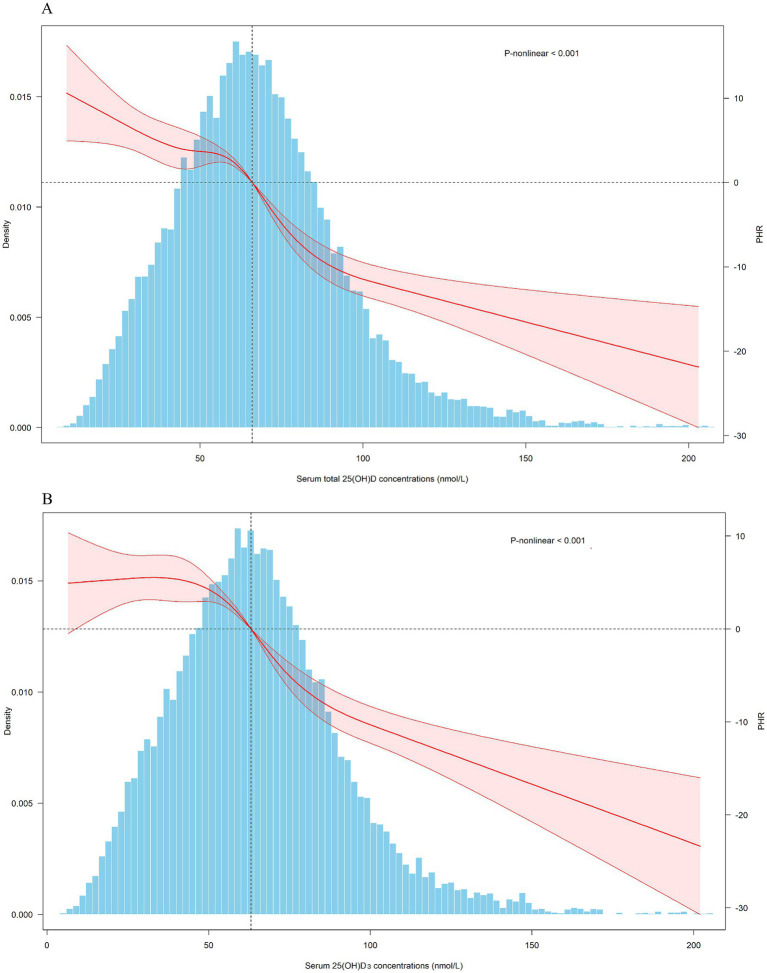
Dose-response relationship of serum 25(OH)D concentrations and PHR. PHR: platelet to high-density lipoprotein cholesterol ratio; CI, confidence interval. Total 25(OH)D: the combined concentrations of 25(OH)D₂ and 25(OH)D₃. The covariates adjusted for are as shown in model 4.

Given the potential non-linear associations between 25(OH)D and PHR, we divided total 25(OH)D into quartiles, using the lowest quartile as the reference group, as presented in Table 2. After adjusting for cycles, we found that an increase in 25(OH)D from Q1 to Q4 was associated with a significant decrease of 28.64 units (95% CI: −31.64 to −25.64) in PHR. After stepwise adjustments for demographic characteristics, lifestyle factors, and health conditions (including medication use), we observed that with increasing 25(OH)D from Q1 to Q4, PHR decreased by 27.65 (95% CI: −31.00 to −24.31), 23.56 (95% CI: −26.99 to −20.12), and 14.84 (95% CI: −18.03 to −11.66) units, respectively. The *P*-trend results further supported the associations observed above. RCS analysis revealed a linear negative association between 25(OH)D levels and PHR (Figure 2). Given that 25(OH)D concentrations are primarily determined by 25(OH)D_3_ levels, we performed similar analyses for 25(OH)D_3_ and found comparable results.

### 3.3 Subgroup and sensitivity analyses

**Figure 3:**
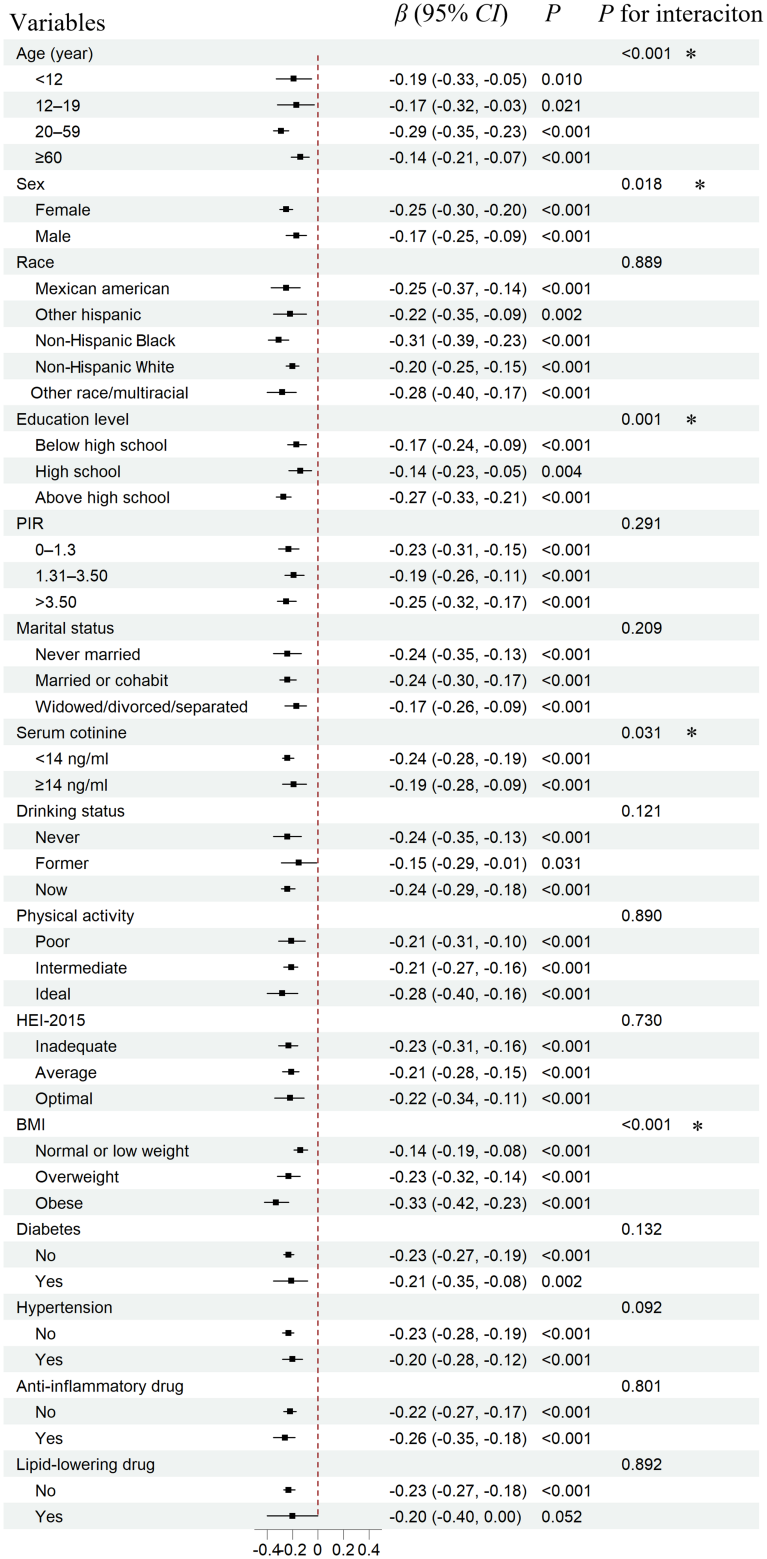
Subgroup analysis for the association between serum total 25(OH)D concentrations and PHR. PHR, platelet to high-density lipoprotein cholesterol ratio; CI, confidence interval. Total 25(OH)D: the combined concentrations of 25(OH)D₂ and 25(OH)D₃. The covariates adjusted for are as shown in model 4. *: *p* < 0.05.

To evaluate the stability of the associations of 25(OH)D with PHR, as well as to identify potentially sensitive subgroups, a subgroup analysis was conducted based on total 25(OH)D levels (Figure 3). Consistent effects with the main findings were observed across nearly all subgroups. Additionally, stronger associations were identified among adults (aged 20–59 years), females, individuals with higher education, non-smokers, and those with a higher BMI. The interaction test further supported these findings, showing statistical significance.

In sensitivity analyses, we first applied general linear regression and observed similar results (Supplementary Table S2). Specifically, in various models, each 1-unit increase in total 25(OH)D was associated with a decrease in PHR ranging from 0.22 to 0.38 units. Additionally, we categorized PHR into a binary variable using the median as the cutoff point and conducted further analysis using weighted logistic regression (Supplementary Table S3). We observed a similar trend. For instance, after stepwise adjustments for demographic characteristics, lifestyle factors, and health conditions (including medication use), each 10-unit increase in total 25(OH)D was correlated with an odds ratio ranging from 0.90 to 0.94. The dose-response analysis of total 25(OH)D concentrations and PHR (as a binary variable) further illustrated the negative association, providing additional support for our primary findings (Supplementary Figure S2). Finally, we categorized 25(OH)D_2_ as a binary variable using the LOD as the cutoff point. The regression coefficients ranged from −3.75 to −12.25 across different models, consistent with the primary findings (Supplementary Table S4).

### 3.4 Validation based on Chinese data

**Figure 4:**
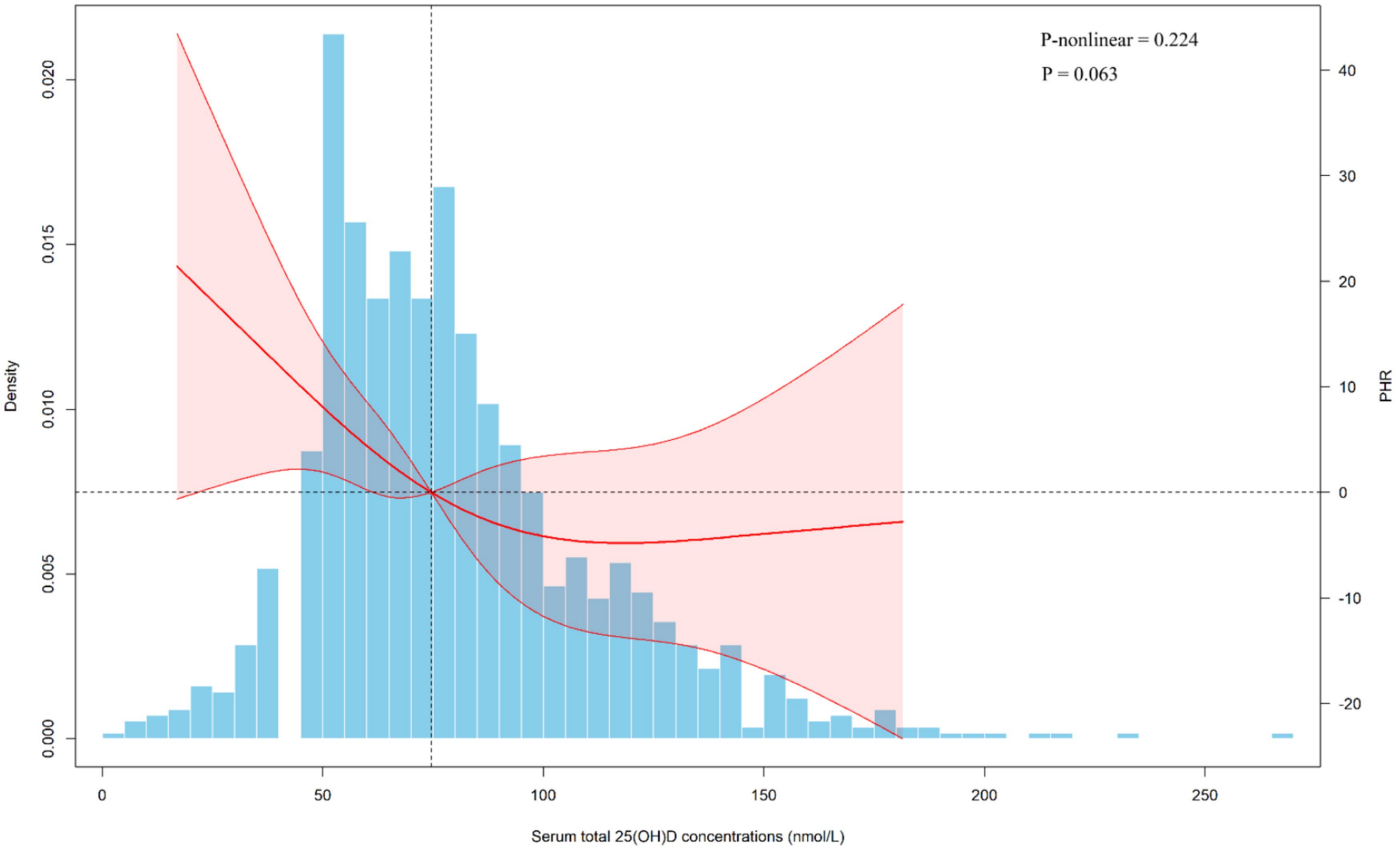
Dose-response relationship of serum total 25(OH)D concentrations and PHR from Chinese adults. PHR, platelet to high-density lipoprotein cholesterol ratio; CI, confidence interval. The covariates adjusted for are as shown in model 4.

Supplementary Table S5 presents the baseline characteristics of the Chinese population and the differences in PHR across subgroups. We observed that individuals who were female, living in poverty, non-drinkers, and those with diabetes or hypertension tended to have higher PHR levels. Linear regression analyses showed associations consistent with those found in the NHANES population (Table 3). Furthermore, restricted cubic spline (RCS) analysis revealed a linear negative association between 25(OH)D levels and PHR (Figure 4). We also performed stratified analyses (Supplementary Table S6). Notably, a significant association between 25(OH)D and PHR was observed among females (*β* = −0.38, 95% CI: −0.62 to −0.14, *p* = 0.002), but not among males (β = −0.07, 95% CI: −0.23 to 0.08, *p* = 0.353), with a significant interaction by sex (P for interaction = 0.034). The findings were in line with the NHANES results.

**Table 3:** Associations of serum total 25(OH)D concentrations with PHR using weighted linear regression from Chinese adults.

Characteristics	Continuous	Q1	Q2	Q3	Q4	P for trend
	*β* (95% *CI*)	Reference	*β* (95% *CI*)	*β* (95% *CI*)	*β* (95% *CI*)	
Model 1	−0.23 (−0.35, −0.11)	1	−12.45 (−23.52, −1.37)	−20.26 (−31.26, −9.25)	−21.45 (−32.60, −10.30)	<0.001
Model 2	−0.18 (−0.31, −0.04)	1	−11.18 (−22.42, 0.06)	−18.01 (−29.40, −6.62)	−16.65 (−29.35, −3.96)	<0.001
Model 3	−0.17 (−0.31, −0.04)	1	−10.35 (−21.59, 0.90)	−17.28 (−28.68, −5.88)	−16.49 (−29.22, −3.77)	<0.001
Model 4	−0.15 (−0.28, −0.01)	1	−10.36 (−21.57, 0.84)	−16.18 (−27.55, −4.81)	−14.83 (−27.54, −2.12)	<0.001

PHR, platelet to high-density lipoprotein cholesterol ratio; CI, confidence interval; Q1, the first quartile; Q2, the second quartile; Q3, the third quartile; Q4, the fourth quartile. Model 1: was unadjusted. Model 2: demographic characteristics (age, sex, education level, economy, and marital status). Model 3: further adjusted for lifestyle factors (smoking status, drinking status, physical activity, pork intake frequency, and fruit intake frequency) in addition to the adjustments in Model 2. Model 4: further adjusted for health conditions and medication use (BMI, diabetes, and hypertension) in addition to the adjustments in Model 3.

## 4 Discussion

### 4.1 Main findings

After stepwise adjustment for cycles, demographic characteristics, lifestyle factors, and health conditions (including medication use), survey-weighted linear regression demonstrated a significant inverse relationship between serum 25(OH)D levels and PHR. Specifically, for every 1-unit increase in 25(OH)D, PHR decreased by 0.23 to 0.41 units across models. Similarly, the association remained significant, with PHR decreasing by 14.84 to 27.65 units in the Q4 of total 25(OH)D compared to the Q1. RCS analysis further supported a linear inverse association between total 25(OH)D and PHR. We also performed similar analyses for 25(OH)D_3_, and the results were generally consistent. Furthermore, analyses in the Chinese population further confirmed the consistent association between serum total 25(OH)D and PHR. Notably, the stronger association observed among females was evident in both populations, with the interaction effect reaching statistical significance.

### 4.2 Comparison with previous work and possible explanations

We included six survey cycles with a weighted PHR range of 189.04 to 219.18, aligning with the ranges reported in previous studies (2, 3, 26). According to the Endocrine Society’s guidelines (31, 32), a serum 25(OH)D concentration of 75 nmol/L (30 ng/mL) or greater is considered sufficient. In this study, we found that 9,747 individuals (26.90%) had 25(OH)D levels of 75 nmol/L or higher. Quartile analysis revealed that Q4 ranged from 76.5 to 215 nmol/L, representing individuals with sufficient 25(OH)D levels to some extent. These findings suggest that the proportion of individuals with sufficient 25(OH)D remains relatively low in the U.S. population. Although the Chinese population demonstrated somewhat better levels, nearly half of the participants still did not reach the 75 nmol/L threshold (Supplementary Table S5).

To our knowledge, only one study using NHANES data has quantified the relationship between 25(OH)D and PHR, and the findings were consistent with ours (24). The previous NHANES study, which included 5,308 participants from 2007 to 2018, reported that each 1-unit increase in 25(OH)D was associated with a 0.17-unit decrease in PHR (95% CI: −0.25 to −0.09) after adjustments (24). In comparison, our analyses in the larger NHANES sample (*n* = 36,238) yielded a similar estimate of −0.23 (95% CI: −0.27 to −0.18), while the Chinese cohort (*n* = 1,122) showed a comparable decrease of −0.15 (95% CI: −0.28 to −0.01). Unlike the previous study, which conducted subgroup analyses only by hypertension and diabetes, we performed stratified analyses across 15 variables. By leveraging two population-based datasets and multiple stratifications, our study further examined the robustness of the association and identified potentially sensitive subpopulations. Additionally, several observational and experimental studies have investigated the relationship between vitamin D and platelet count as well as HDL cholesterol, and their results were consistent with our findings (23, 33–35).

In the study conducted by Park et al. (33), which included 3,190 adults aged over 20 years, they found a significant negative correlation between vitamin D levels and platelet count. In another study, which included 341 overweight and obese individuals aged 18–71 years, they observed that sufficient vitamin D levels were independently associated with lower platelet counts (34). Other studies also have indicated a possible association between vitamin D insufficiency and elevated platelet counts (19, 20). The inverse relationship between vitamin D levels and platelet count is biologically plausible, as vitamin D exerts anti-inflammatory and antithrombotic effects (36). Specifically, vitamin D has been shown to modulate inflammatory and coagulation pathways, including its impact on endothelial cell activation (18).

A longitudinal community-based study of 13,039 participants showed that adequate 25(OH)D concentrations were prospectively associated with higher HDL-C levels (23). In addition, Faridi et al. (23) and Lupton et al. (17) both reported a significant correlation between adequate vitamin D levels and higher HDL-C levels. In contrast, a cross-sectional study at the polycystic ovary syndrome and infertility clinic of Arash Women’s Hospital in Tehran found no effect of vitamin D deficiency on the lipid profile (35). These differences may largely be attributed to variations in geographic factors, demographic characteristics, and study design. In general, however, vitamin D appears to be positively associated with HDL-C levels. The relationship between vitamin D and elevated HDL-C levels is biologically plausible. Vitamin D may influence cholesterol synthesis and transport by regulating key enzymes involved in lipid metabolism in the liver (21, 22). These mechanisms could contribute to increased HDL synthesis (21, 22).

### 4.3 Subgroup analysis

Subgroup analyses revealed consistent results with the main findings across nearly all subgroups. This consistency suggests that the observed effects are not substantially influenced by the potential confounders measured in this study, such as the use of anti-inflammatory or lipid-lowering medications, further reinforcing the stability and reliability of our results. NHANES data revealed that the inverse association between serum 25(OH)D and PHR was more pronounced among adults aged 20–59 years, females, individuals with higher educational attainment, non-smokers, and those with elevated BMI, with a statistically significant interaction (*p* < 0.05). Importantly, we replicated the stronger female-specific association in the Chinese cohort, with a statistically significant interaction (*p* = 0.034). This sex-difference may reflect underlying biological mechanisms, such as hormonal regulation of vitamin D metabolism and platelet activity, as well as gender-specific lifestyle factors (37, 38). Estrogen has been shown to increase the expression of vitamin D receptors, thereby enhancing the biological activity of vitamin D in females (39). Moreover, a review suggested that estrogen can further enhance vitamin D function, promote its accumulation, and increase receptor expression, leading to more effective anti-inflammatory responses in females than in males (37). Together, these mechanisms may explain the observed gender differences in vitamin D metabolism and its impact on health outcomes. Although definitive conclusions are premature, this sex-specific disparity merits further investigation and could inform the design of tailored public health strategies for at-risk populations.

### 4.4 Strengths and limitations

This study has two major strengths. First, this study represents the first combined analysis of data from the U.S. and China examining the relationship between vitamin D levels and PHR, and is also the first to reveal a possible heightened sensitivity to this association in females. Second, the study employed a range of statistical methods and adjusted for as many potential confounders as possible, thereby improving the robustness and reliability of the results. Admittedly, this study has several limitations. First, since both datasets are cross-sectional, this study cannot establish the temporal relationship between serum 25(OH)D levels and blood PHR, and therefore cannot confirm causality. However, the findings provide preliminary evidence, paving the way for future longitudinal and experimental studies to further explore these associations. Second, serum 25(OH)D levels were measured using different methods in the two studies, which may introduce measurement bias. Additionally, although we attempted to harmonize covariates across the two datasets as much as possible, some differences in definitions and limitations in data availability remained. Finally, although we adjusted for many potential confounders in the analyses, unmeasured confounders, such as environmental exposures like air pollution, cannot be entirely ruled out.

## 5 Conclusion

Our study found a significant inverse association between total serum 25(OH)D levels and PHR. The association was particularly stronger in females, suggesting they may be more sensitive to this association. These findings imply that 25(OH)D may play a role in modulating PHR levels, potentially helping to prevent or mitigate the progression of related diseases. Future research should adopt longitudinal and experimental designs to confirm causality and investigate the underlying mechanisms, paving the way for potential clinical applications.

## Data Availability

The datasets presented in this article are not readily available because the Chinese datasets used and/or analyzed in this study are available from the corresponding author upon reasonable request. Requests to access the datasets should be directed to JZ, wokey2001@163.com.

## Footnotes

^1^ https://www.cdc.gov/nchs/nhanes/about
