# Supplementary material for: Association between serum 25-hydroxyvitamin D concentrations and platelet to high-density lipoprotein cholesterol ratio: evidence from two population-based studies
Source: Front Nutr. 2025 Oct 6;12:1662753. doi: 10.3389/fnut.2025.1662753 (PMC12536551; doi:10.3389/fnut.2025.1662753)
Supplement: Supplementary file 1 [file Data_Sheet_1.docx]

**Supplementary materials**

**Table S1** Characteristics of the original NHANES population and the included/excluded population (n, %).

**Table S2** Associations of serum 25(OH)D concentrations with PHR using general linear regression.

**Table S3** Associations of serum 25(OH)D concentrations (per 10-unit increase) with PHR (<186.93 and ≥186.93) using weighted logistic regression.

**Table S4** Associations of serum 25(OH)D_2_ concentrations with PHR using weighted linear regression.

**Table S5** The characteristics of all eligible participants from Chinese adults.

**Table S6** Subgroup analysis for the association between serum total 25(OH)D concentrations and PHR from Chinese adults.

**Fig. S1** Histogram of PHR and serum 25(OH)D concentrations in this study.

**Fig. S2** Dose-response relationship of serum 25(OH)D concentrations and PHR.

**Table S1** Characteristics of the original NHANES population and the included/excluded population (n, %).

| Variables | Original  Population | Inclusion of population | Exclusion of population |
| --- | --- | --- | --- |
|  | (n = 42,744) ^a^ | (n = 36,238) | (n = 6,506) |
| Cycles |  |  |  |
| 2007–2008 | 6385 (14.00) | 5559 (14.46) | 826 (11.46) |
| 2009–2010 | 7821 (16.47) | 6858 (16.89) | 963 (14.12) |
| 2011–2012 | 6979 (16.86) | 5944 (17.01) | 1035 (16.08) |
| 2013–2014 | 7613 (17.52) | 6318 (17.15) | 1295 (19.59) |
| 2015–2016 | 7214 (17.41) | 6069 (17.17) | 1145 (18.73) |
| 2017–2018 | 6732 (17.74) | 5490 (17.32) | 1242 (20.02) |
| Age (year) |  |  |  |
| <12 | 5638 (7.19) | 4958 (7.52) | 680 (5.38) |
| 12–19 | 6534 (11.28) | 6158 (12.64) | 376 (3.83) |
| 20–59 | 20173 (59.79) | 17632 (61.88) | 2541 (48.32) |
| ≥60 | 10399 (21.73) | 7490 (17.96) | 2909 (42.47) |
| Sex |  |  |  |
| Female | 21692 (51.18) | 18133 (50.07) | 3559 (57.28) |
| Male | 21052 (48.82) | 18105 (49.93) | 2947 (42.72) |
| Race/ethnicity |  |  |  |
| Mexican American | 7553 (10.00) | 6685 (10.46) | 868 (7.48) |
| Other Hispanic | 4638 (6.27) | 4027 (6.43) | 611 (5.35) |
| Non-Hispanic black | 9169 (11.24) | 7961 (11.60) | 1208 (9.21) |
| Non-Hispanic white | 15986 (64.27) | 13128 (63.44) | 2858 (68.82) |
| Other race/multiracial | 5398 (8.23) | 4437 (8.06) | 961 (9.15) |
| Education level |  |  |  |
| Below high school | 18635 (29.53) | 16275 (30.70) | 2360 (23.11) |
| High school | 7539 (19.74) | 6317 (19.88) | 1222 (18.96) |
| Above high school | 16528 (50.67) | 13646 (49.42) | 2882 (57.53) |
| Missing | 42 (0.06) | 0 (0.00) | 42 (0.40) |
| PIR |  |  |  |
| Low (0–1.3) | 14075 (22.41) | 12105 (22.78) | 1970 (20.36) |
| Middle (1.31–3.50) | 14395 (33.14) | 12263 (33.54) | 2132 (30.99) |
| High (>3.50) | 10484 (37.21) | 8859 (37.02) | 1625 (38.22) |
| Missing | 3790 (7.24) | 3011 (6.66) | 779 (10.43) |
| Marital status |  |  |  |
| Never married | 5553 (14.78) | 4878 (15.54) | 675 (10.63) |
| Married or cohabit | 18190 (51.69) | 14962 (50.61) | 3228 (57.67) |
| Widowed/divorced/separated | 6813 (15.02) | 5276 (13.67) | 1537 (22.43) |
| Missing | 12188 (18.50) | 11122 (20.18) | 1066 (9.28) |
| Serum cotinine |  |  |  |
| <14 ng/ml | 34804 (79.48) | 29530 (79.48) | 5274 (79.43) |
| ≥14 ng/ml | 7895 (20.44) | 6708 (20.52) | 1187 (20.02) |
| Missing | 45 (0.08) | 0 (0.00) | 45 (0.55) |
| Drinking status |  |  |  |
| Never | 4315 (8.64) | 3621 (8.58) | 694 (9.00) |
| Former | 4258 (9.32) | 3463 (8.97) | 795 (11.23) |
| Now | 19366 (56.90) | 16825 (58.27) | 2541 (49.32) |
| Missing | 14805 (25.14) | 12329 (24.18) | 2476 (30.45) |
| Physical activity |  |  |  |
| Low | 4779 (11.63) | 3982 (11.40) | 797 (12.89) |
| Moderate | 18697 (50.65) | 16362 (52.13) | 2335 (42.51) |
| Optimal | 3867 (9.71) | 3227 (9.50) | 640 (10.85) |
| Missing | 15401 (28.01) | 12667 (26.97) | 2734 (33.75) |
| HEI-2015 |  |  |  |
| Inadequate | 21135 (49.58) | 19554 (53.45) | 1581 (28.29) |
| Average | 15466 (36.76) | 13977 (38.60) | 1489 (26.62) |
| Optimal | 3110 (7.87) | 2707 (7.95) | 403 (7.42) |
| Missing | 3033 (5.79) | 0 (0.00) | 3033 (37.66) |
| BMI |  |  |  |
| Normal or low weight | 17913 (37.90) | 15612 (38.94) | 2301 (32.18) |
| Overweight | 11619 (29.17) | 9805 (28.99) | 1814 (30.15) |
| Obese | 12730 (31.99) | 10821 (32.07) | 1909 (31.57) |
| Missing | 482 (0.94) | 0 (0.00) | 482 (6.10) |
| Diabetes |  |  |  |
| No | 37905 (90.17) | 32745 (91.92) | 5160 (80.54) |
| Yes | 4519 (8.95) | 3493 (8.08) | 1026 (13.74) |
| Missing | 320 (0.88) | 0 (0.00) | 320 (5.72) |
| Hypertension |  |  |  |
| No | 27849 (67.32) | 24491 (69.70) | 3358 (54.18) |
| Yes | 12842 (30.07) | 10039 (27.72) | 2803 (42.99) |
| Missing | 2053 (2.62) | 1708 (2.58) | 345 (2.83) |
| Anti-inflammatory drug |  |  |  |
| No | 36303 (84.54) | 31426 (86.42) | 4877 (74.19) |
| Yes | 6417 (15.41) | 4812 (13.58) | 1605 (25.48) |
| Missing | 24 (0.05) | 0 (0.00) | 24 (0.33) |
| Lipid-lowering drug |  |  |  |
| No | 40889 (95.50) | 34757 (95.83) | 6132 (93.72) |
| Yes | 1831 (4.45) | 1481 (4.17) | 350 (5.96) |
| Missing | 24 (0.05) | 0 (0.00) | 24 (0.33) |

Abbreviations: n: numbers of subjects; %: weighted percentage; PIR: poverty-to-income ratio; HEI: healthy eating index; BMI: body mass index.

^a^: Population with complete data on serum 25(OH)D concentrations and PHR.

**Table S2** Associations of serum 25(OH)D concentrations with PHR using general linear regression.

| Characteristics | Continuous | Q1 | Q2 | Q3 | Q4 | *P* for trend |
| --- | --- | --- | --- | --- | --- | --- |
|  | *β* (95% *CI*) | Reference | *β* (95% *CI*) | *β* (95% *CI*) | *β* (95% *CI*) |  |
| Total 25(OH)D |  |  |  |  |  |  |
| Model 1 | -0.38 ( -0.41, -0.35) | 1.00 | 0.24 (-1.95, 2.44) | -6.61 (-8.81, -4.40) | -20.3 (-22.50, -18.10) | <0.001 |
| Model 2 | -0.37 ( -0.40, -0.33) | 1.00 | -6.37 (-8.58, -4.16) | -14.27 (-16.56, -11.98) | -23.37 (-25.75, -20.99) | <0.001 |
| Model 3 | -0.33 (-0.37, -0.30) | 1.00 | -5.2 (-7.41, -3.00) | -12.62 (-14.90, -10.33) | -20.8 (-23.19, -18.41) | <0.001 |
| Model 4 | -0.22 (-0.25, -0.18) | 1.00 | -2.82 (-4.93, -0.71) | -7.75 (-9.94, -5.55) | -12.83 (-15.14, -10.52) | <0.001 |
| 25(OH)D_3_ |  |  |  |  |  |  |
| Model 1 | -0.35 (-0.38, -0.32) | 1.00 | 0.24 (-1.95, 2.44) | -6.61 (-8.81, -4.40) | -20.3 (-22.50, -18.10) | <0.001 |
| Model 2 | -0.37 (-0.41, -0.34) | 1.00 | -6.37 (-8.58, -4.16) | -14.27 (-16.56, -11.98) | -23.37 (-25.75, -20.99) | <0.001 |
| Model 3 | -0.33 (-0.37, -0.30) | 1.00 | -5.2 ( -7.41, -3.00) | -12.62 (-14.90, -10.33) | -20.8 (-23.19, -18.41) | <0.001 |
| Model 4 | -0.21 (-0.24, -0.17) | 1.00 | -2.82 (-4.93, -0.71) | -7.7 5 -9.94, -5.55) | -12.83 (-15.14, -10.52) | <0.001 |

Abbreviations: PHR: platelet to highdensity lipoprotein cholesterol ratio; CI, confidence interval; Q1, the first quartile; Q2, the second quartile; Q3, the third quartile; Q4, the fourth quartile. Total 25(OH)D: the combined concentrations of 25(OH)D₂ and 25(OH)D₃. Model 1: was adjusted for cycles. Model 2: cycles and demographic characteristics (age, sex, race, education level, PIR, and marital status). Model 3: further adjusted for lifestyle factors (serum cotinine, drinking status, physical activity, and HEI-2015) in addition to the adjustments in Model 2. Model 4: further adjusted for health conditions and medication use (BMI, diabetes, hypertension, anti-inflammatory drugs, and lipid-lowering drugs) in addition to the adjustments in Model 3.

**Table S3** Associations of serum 25(OH)D concentrations (per 10-unit increase) with PHR (<186.93 and ≥186.93) using weighted logistic regression.

| Characteristics | Continuous | Q1 | Q2 | Q3 | Q4 | *P* for trend |
| --- | --- | --- | --- | --- | --- | --- |
|  | *OR* (95% *CI*) | Reference | *OR* (95% *CI*) | *OR* (95% *CI*) | *OR* (95% *CI*) |  |
| Total 25(OH)D |  |  |  |  |  |  |
| Model 1 | 0.90 (0.88, 0.91) | 1.00 | 1.08 (0.99, 1.17) | 0.88 (0.81, 0.95) | 0.58 (0.54, 0.63) | <0.001 |
| Model 2 | 0.90 (0.89,0.92) | 1.00 | 0.94 (0.87, 1.02) | 0.76 (0.70, 0.83) | 0.56 (0.51, 0.61) | <0.001 |
| Model 3 | 0.91 (0.90,0.93) | 1.00 | 0.98 (0.90, 1.07) | 0.81 (0.73, 0.89) | 0.61 (0.56, 0.67) | <0.001 |
| Model 4 | 0.94 (0.93,0.95) | 1.00 | 1.04 (0.95, 1.13) | 0.92 (0.84, 1.01) | 0.75 (0.69, 0.83) | <0.001 |
| 25 (OH)D_3_ |  |  |  |  |  |  |
| Model 1 | 0.90 (0.89, 0.91) | 1.00 | 1.08 (0.99, 1.17) | 0.88 (0.81, 0.95) | 0.58 (0.54, 0.63) | <0.001 |
| Model 2 | 0.90 (0.89, 0.91) | 1.00 | 0.94 (0.87, 1.02) | 0.76 (0.70, 0.83) | 0.56 (0.51, 0.61) | <0.001 |
| Model 3 | 0.91 (0.90, 0.93) | 1.00 | 0.98 (0.90, 1.07) | 0.81 (0.73, 0.89) | 0.61 (0.56, 0.67) | <0.001 |
| Model 4 | 0.94 (0.93, 0.96) | 1.00 | 1.04 (0.95, 1.13) | 0.92 (0.84, 1.01) | 0.75 (0.69, 0.83) | <0.001 |

Abbreviations: PHR: platelet to highdensity lipoprotein cholesterol ratio; CI, confidence interval; Q1, the first quartile; Q2, the second quartile; Q3, the third quartile; Q4, the fourth quartile. Total 25(OH)D: the combined concentrations of 25(OH)D₂ and 25(OH)D₃. Model 1: was adjusted for cycles. Model 2: cycles and demographic characteristics (age, sex, race, education level, PIR, and marital status). Model 3: further adjusted for lifestyle factors (serum cotinine, drinking status, physical activity, and HEI-2015) in addition to the adjustments in Model 2. Model 4: further adjusted for health conditions and medication use (BMI, diabetes, hypertension, anti-inflammatory drugs, and lipid-lowering drugs) in addition to the adjustments in Model 3.

**Table S4** Associations of serum 25(OH)D_2_ concentrations with PHR using weighted linear regression.

| Characteristics | Low (<LOD) | High (≥LOD) |
| --- | --- | --- |
|  | Reference | *β* (95% *CI*) |
| Model 1 | 1.00 | -12.25 (-14.99, -9.50) |
| Model 2 | 1.00 | -5.98 (-8.70, -3.26) |
| Model 3 | 1.00 | -4.91 (-7.52, -2.30) |
| Model 4 | 1.00 | -3.75 (-6.27, -1.23) |

Abbreviations: PHR: platelet to highdensity lipoprotein cholesterol ratio; LOD: limit of detection. CI, confidence interval. In this study, the detection rate for 25(OH)D_2_ was 17.62%. Model 1: was adjusted for cycles. Model 2: cycles and demographic characteristics (age, sex, race, education level, PIR, and marital status). Model 3: further adjusted for lifestyle factors (serum cotinine, drinking status, physical activity, and HEI-2015) in addition to the adjustments in Model 2. Model 4: further adjusted for health conditions and medication use (BMI, diabetes, hypertension, anti-inflammatory drugs, and lipid-lowering drugs) in addition to the adjustments in Model 3.

**Table S5** The characteristics of all eligible participants from Chinese adults

| Variables | Total | PHR | F/t value | *P* value |
| --- | --- | --- | --- | --- |
|  | n (%) | Mean ± SD |  |  |
| Age (year) |  |  |  |  |
| 60–69 | 524 (46.70) | 155.96 ± 68.22 | 0.49 | 0.622 |
| ≥70 | 598 (53.30) | 153.97 ± 66.86 |  |  |
| Sex |  |  |  |  |
| Female | 571 (50.89) | 161.11 ± 71.04 | -3.15 | 0.002 |
| Male | 551 (49.11) | 148.48 ± 63.01 |  |  |
| Education level |  |  |  |  |
| No formal education | 602 (53.65) | 153.90 ± 67.18 | 0.14 | 0.937 |
| Primary school | 169 (15.06) | 157.55 ± 66.23 |  |  |
| Junior high school | 155 (13.81) | 154.97 ± 71.80 |  |  |
| Above high school | 196 (17.47) | 155.65 ± 66.38 |  |  |
| Economy |  |  |  |  |
| Poverty | 121 (10.78) | 155.79 ± 67.15 | 6.12 | 0.002 |
| Average or wealthy | 478 (42.60) | 147.04 ± 61.38 |  |  |
| Missing | 523 (46.61) | 161.89 ± 72.07 |  |  |
| Marital status |  |  |  |  |
| With spouse | 1044 (93.05) | 155.76 ± 67.27 | 2.41 | 0.121 |
| Without spouse | 78 (6.95) | 143.47 ± 69.62 |  |  |
| Smoking status |  |  |  |  |
| Never smoked | 783 (69.79) | 156.39 ± 67.30 | 0.65 | 0.523 |
| Former smoker | 132 (11.76) | 152.44 ± 69.19 |  |  |
| Currently smoking | 207 (18.45) | 150.86 ± 67.17 |  |  |
| Drinking status |  |  |  |  |
| Non-drinker | 874 (77.90) | 157.91 ± 69.72 | 3.33 | 0.019 |
| 1–4 times per month | 26 (2.32) | 152.55 ± 46.39 |  |  |
| 1–6 times per week | 77 (6.86) | 151.33 ± 58.49 |  |  |
| Daily drinker | 145 (12.92) | 139.11 ± 59.07 |  |  |
| Physical activity |  |  |  |  |
| Low | 696 (62.03) | 158.47 ± 69.93 | 2.75 | 0.064 |
| Moderate | 356 (31.73) | 148.19 ± 63.34 |  |  |
| Optimal | 70 (6.24) | 153.63 ± 60.96 |  |  |
| Pork intake frequency |  |  |  |  |
| Never | 227 (20.23) | 152.52 ± 63.22 | 0.35 | 0.786 |
| 1–4 times per month | 331 (29.50) | 154.86 ± 64.99 |  |  |
| 1–6 times per week | 468 (41.71) | 156.93 ± 69.76 |  |  |
| Every day | 96 (8.56) | 150.78 ± 74.71 |  |  |
| Fruit intake frequency |  |  |  |  |
| Never | 173 (15.42) | 157.87 ± 63.43 | 0.71 | 0.548 |
| 1–4 times per month | 144 (12.83) | 160.77 ± 65.81 |  |  |
| 1–6 times per week | 481 (42.87) | 152.35 ± 68.84 |  |  |
| Every day | 324 (28.88) | 154.50 ± 68.35 |  |  |
| BMI |  |  |  |  |
| Normal or low weight | 505 (45.01) | 150.32 ± 70.66 | 2.45 | 0.087 |
| Overweight | 410 (36.54) | 160.21 ± 65.11 |  |  |
| Obese | 207 (18.45) | 155.57 ± 63.54 |  |  |
| Diabetes |  |  |  |  |
| No | 941 (83.87) | 151.99 ± 67.83 | -3.32 | 0.001 |
| Yes | 181 (16.13) | 170.07 ± 63.70 |  |  |
| Hypertension |  |  |  |  |
| No | 445 (39.66) | 149.06 ± 67.52 | -2.36 | 0.019 |
| Yes | 677 (60.34) | 158.75 ± 67.23 |  |  |
| Total 25(OH)D (nmol/L) |  |  |  |  |
| Q1 (4.4–57.0) | 281 (25.0) | 167.62 ± 71.71 | 5.91 | 0.001 |
| Q2 (57.1–74.6) | 281 (25.0) | 157.36 ± 71.08 |  |  |
| Q3 (74.7–95.7) | 280 (25.0) | 147.47 ± 65.07 |  |  |
| Q4 (95.8–562.2) | 280 (25.0) | 147.10 ± 59.53 |  |  |

Abbreviations: PHR: platelet to high-density lipoprotein cholesterol ratio; SD: standard deviation; BMI: body mass index.

**Table S6** Subgroup analysis for the association between serum total 25(OH)D concentrations and PHR from Chinese adults

| Variables | *β* (95% *CI*) | *P* value | *P* for interaction |
| --- | --- | --- | --- |
| Age (year) |  |  |  |
| 60–69 | -0.20 (-0.39, -0.01) | 0.038 | 0.724 |
| ≥70 | -0.24 (-0.40, -0.09) | 0.002 |  |
| Sex |  |  |  |
| Female | -0.38 (-0.62, -0.14) | 0.002 | 0.034* |
| Male | -0.07 (-0.23, 0.08) | 0.353 |  |
| Education level |  |  |  |
| No formal education | -0.22 (-0.39, -0.05) | 0.011 | 0.925 |
| Primary school | -0.28 (-0.61, 0.04) | 0.090 |  |
| Junior high school | -0.28 (-0.61, 0.04) | 0.089 |  |
| Above high school | -0.20 (-0.49, 0.08) | 0.160 |  |
| Economy |  |  |  |
| Poverty | -0.24 (-0.57, 0.10) | 0.169 | 0.008* |
| Average or wealthy | 0.00 (-0.17, 0.16) | 0.952 |  |
| Missing | -0.44 (-0.68, -0.19) | 0.001 |  |
| Marital status |  |  |  |
| With spouse | -0.24 (-0.37, -0.12) | <0.001 | 0.613 |
| Without spouse | -0.09 (-0.70, 0.53) | 0.780 |  |
| Smoking status |  |  |  |
| Never smoked | -0.29 (-0.44, -0.14) | <0.001 | 0.223 |
| Former smoker | -0.15 (-0.50, 0.21) | 0.429 |  |
| Currently smoking | -0.06 (-0.33, 0.21) | 0.670 |  |
| Drinking status |  |  |  |
| Non-drinker | -0.29 (-0.43, -0.15) | <0.001 | 0.021* |
| 1–4 times per month | 0.09 (-0.56, 0.73) | 0.795 |  |
| 1–6 times per week | 0.15 (-0.21, 0.50) | 0.419 |  |
| Daily drinker | 0.04 (-0.27, 0.35) | 0.812 |  |
| Physical activity |  |  |  |
| Low | -0.24 (-0.39, -0.09) | 0.002 | 0.595 |
| Moderate | -0.23 (-0.46, -0.01) | 0.041 |  |
| Optimal | -0.11 (-0.55, 0.32) | 0.616 |  |
| Pork intake frequency |  |  |  |
| Never | -0.22 (-0.47, 0.03) | 0.086 | 0.816 |
| 1–4 times per month | -0.29 (-0.50, -0.08) | 0.006 |  |
| 1–6 times per week | -0.17 (-0.36, 0.03) | 0.090 |  |
| Every day | -0.38 (-0.94, 0.17) | 0.177 |  |
| Fruit intake frequency |  |  |  |
| Never | -0.31 (-0.58, -0.04) | 0.027 | 0.920 |
| 1–4 times per month | -0.02 (-0.35, 0.31) | 0.912 |  |
| 1–6 times per week | -0.25 (-0.44, -0.05) | 0.015 |  |
| Every day | -0.25 (-0.47, -0.03) | 0.028 |  |
| BMI |  |  |  |
| Normal or low weight | -0.33 (-0.51, -0.15) | <0.001 | 0.136 |
| Overweight | -0.05 (-0.25, 0.16) | 0.646 |  |
| Obese | -0.23 (-0.50, 0.05) | 0.106 |  |
| Diabetes |  |  |  |
| No | -0.23 (-0.36, -0.10) | 0.001 | 0.411 |
| Yes | -0.08 (-0.40, 0.24) | 0.623 |  |
| Hypertension |  |  |  |
| No | -0.33 (-0.51, -0.15) | <0.001 | 0.094 |
| Yes | -0.12 (-0.29, 0.04) | 0.136 |  |

Abbreviations: CI, confidence interval. The covariates adjusted for are as shown in model 4. *: P<0.05.


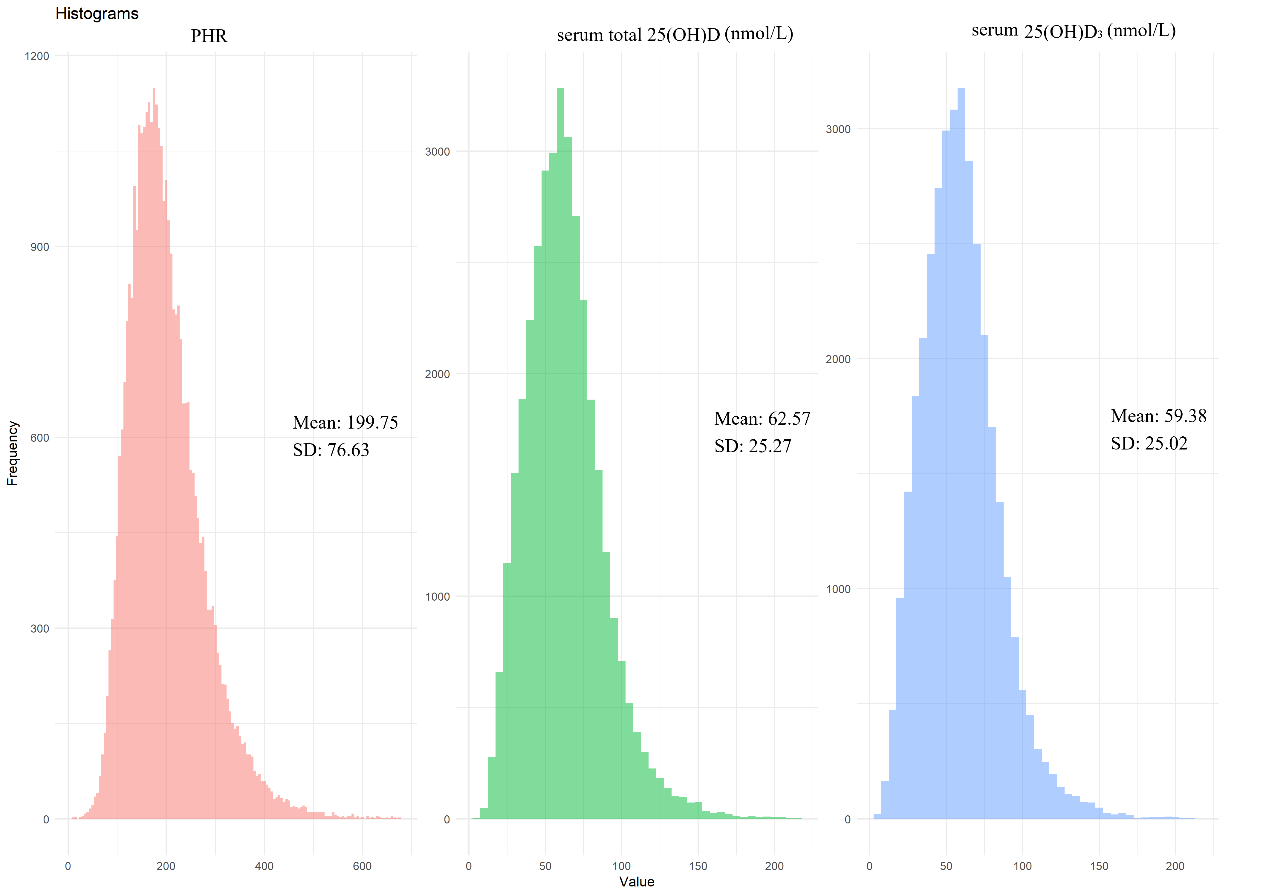
 **Fig. S1** Histogram of PHR and serum 25(OH)D concentrations in this study.


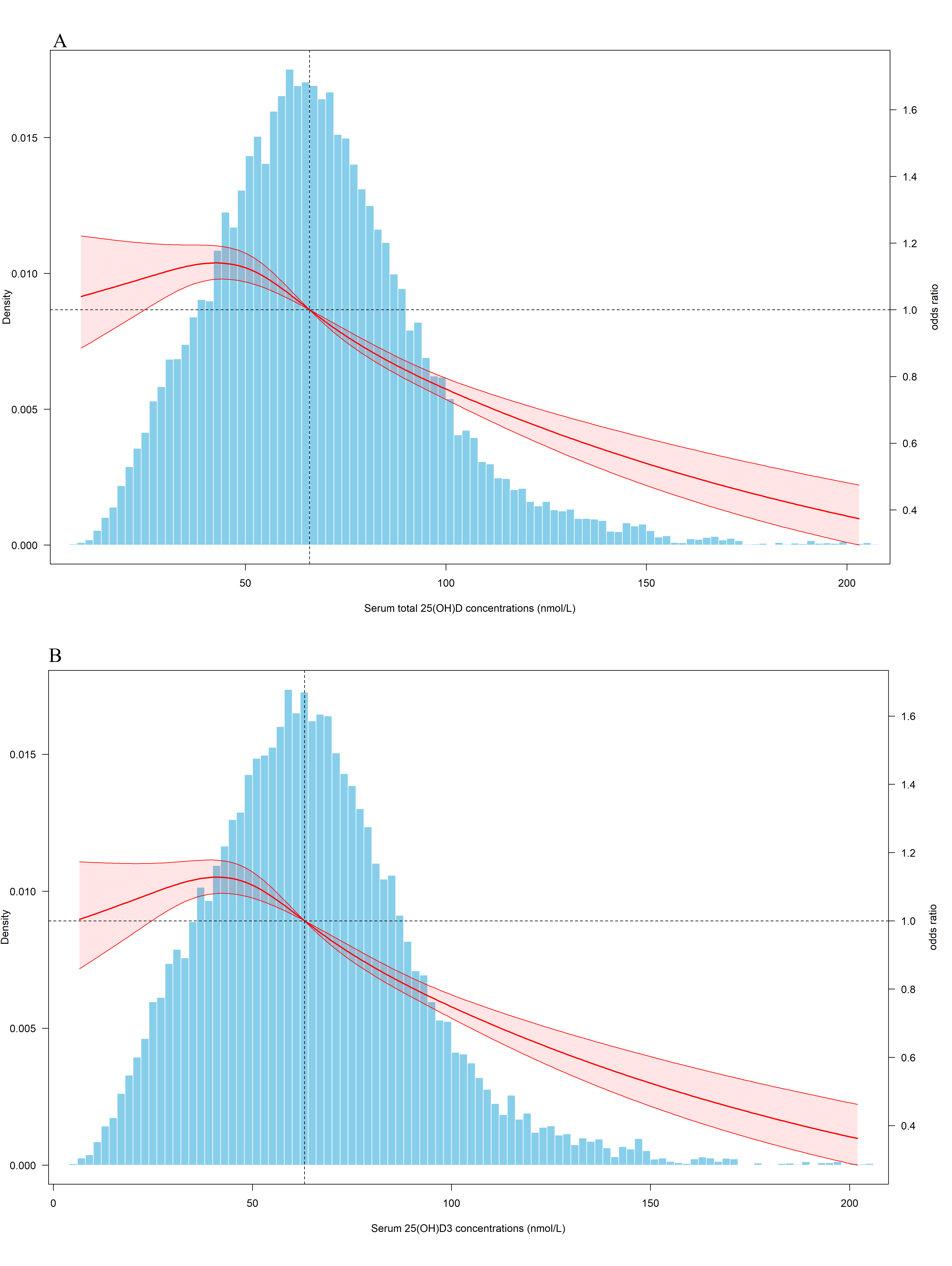
**Fig. S2** Dose-response relationship of serum 25(OH)D concentrations and PHR. Abbreviations: PHR: platelet to highdensity lipoprotein cholesterol ratio; CI, confidence interval. Total 25(OH)D: the combined concentrations of 25(OH)D₂ and 25(OH)D₃. The covariates adjusted for are as shown in model 4.
